# Placental endothelial nitric oxide synthase expression and role of oxidative stress in susceptibility to preeclampsia in Pakistani women

**DOI:** 10.1002/mgg3.1191

**Published:** 2020-03-05

**Authors:** Ghazala Shaheen, Sarwat Jahan, Qurat Ul Ain, Asad Ullah, Tayyaba Afsar, Ali Almajwal, Iftikhar Alam, Suhail Razak

In Shaheen et al. (2019), the address reflected for the corresponding author, Suhail Razak, in the Correspondence section was incorrect. The correct information is shown below.

**Correspondence**

Suhail Razak, Community Health Sciences, College of Applied Medical Sciences, King Saud University, Riyadh, KSA

Emails: ruhail12345@yahoo.com; smarazi@ksu.edu.sa
